# Challenges Encountered in the Provision of Enteral Nutrition in Pediatric Intensive Care Unit: An Observational Study

**DOI:** 10.7759/cureus.49285

**Published:** 2023-11-23

**Authors:** Mohammad Haseeb, Mahafrin H Goiporia, Mohd Saeed Siddiqui, Avinash L Sangle, Syed F Quadri, Ramula L Ravali

**Affiliations:** 1 Department of Pediatrics, Mahatma Gandhi Mission's (MGM) Medical College and Hospital, a Constituent Unit of MGM Institute of Health Sciences, Aurangabad, IND; 2 Department of Public Health, John Snow, Inc. (JSI), Delhi, IND

**Keywords:** delayed nutrition, nutritional support, en interruptions, pediatric intensive care units, enteral nutrition

## Abstract

Background

Enteral nutrition (EN) represents the preferred modality for nutrient administration in critically ill patients. However, it is fraught with challenges such as delayed initiation and recurrent interruptions, which can significantly impact patient clinical outcomes. A substantial proportion of these interruptions can be mitigated. In the present investigation, our objective was to scrutinize the practice of EN in the Pediatric Intensive Care Unit (PICU). We sought to ascertain the frequency and underlying causes of EN interruptions and assess their ramifications for nutrient delivery.

Study design

We conducted an observational study within the PICU of Mahatma Gandhi Mission's (MGM) Medical College and Hospital, Aurangabad. The study encompassed children admitted to the PICU for a period exceeding 24 hours who were receiving enteral feeds. We documented the time of commencing EN from the point of PICU admission, instances of enteral feeding interruptions, the number and duration of each interruption episode, and the reasons behind these interruptions. Subsequently, we categorized the causes of feeding interruptions into avoidable and non-avoidable determinants.

Results

Out of the 100 patients enrolled in this study, only 34% presented with normal nutritional status upon admission. Sixty-five percent of patients had their nutritional support initiated within the first 48 hours of admission to the PICU. The median duration from PICU admission to the initiation of EN was 32.5 hours, with a median interruption duration per patient of 40.96 hours. Common causes of interruptions included radiological procedures, respiratory distress, altered sensorium, presence of gastric aspirates, and surgical procedures. Upon analysis, it was determined that a substantial majority, constituting 74%, of these interruptions were avoidable.

Conclusions

The primary challenges associated with EN in the PICU encompass delayed initiation of enteral feeds and frequent interruptions. Importantly, a significant proportion of these issues are avoidable.

## Introduction

A substantial portion of children admitted to critical care units exhibit malnourishment upon admission. Many of these patients experience further deterioration during treatment due to the metabolic response to injury, surgery, or illness [1,2]. Effective nutritional support during the Pediatric Intensive Care Unit (PICU) stay can mitigate nutrient deficits and prevent the development of malnutrition [3]. Enteral nutrition (EN) is the preferred method for administering nutrients to critically ill patients with intact gastrointestinal function, given its cost-effectiveness and lower complication rates in comparison to parenteral nutrition. Nevertheless, EN is associated with several challenges, including delays in initiation, inadequate escalation of feeding rates, frequent interruptions, and failure to achieve energy targets. Once EN is successfully initiated during critical illness, it is often discontinued temporarily for diagnostic or therapeutic procedures. Frequent interruptions in enteral nutrient delivery have a detrimental impact on clinical outcomes due to suboptimal calorie provision [4]. Many of these periprocedural disruptions can be influenced and, importantly, prevented [4,5]. This study aimed to evaluate EN practices within the PICU, focusing on the time of initiation of enteral feeds, the frequency and reasons for EN interruptions, and their implications during critical illness.

## Materials and methods

Study design

An observational and cross-sectional study was carried out at Mahatma Gandhi Mission's (MGM) Medical College and Hospital, a tertiary care center in Aurangabad (Maharashtra, India), from October 2019 to September 2021.

Study participants

The study was conducted within the confines of the 20-bed PICU situated at MGM Medical College and Hospital in Aurangabad. This facility serves as a tertiary care referral hospital, where critically ill pediatric patients presenting with a spectrum of medical and surgical conditions are routinely admitted for intensive care.

It is notable that the management of EN within this PICU does not strictly adhere to a formalized protocol. Instead, the determination and decisions pertaining to enteral feeding are made under the purview of the attending pediatric intensivists, who exercise their clinical judgment in guiding EN practices. The intermittent bolus feeding method was used for giving EN.

Inclusion and exclusion criteria

This study considered eligible participants as infants and children aged between 1 month and 18 years who had been admitted to the PICU for a duration exceeding 24 hours and were receiving intermittent bolus enteral feedings. Patients with malabsorption syndromes were excluded.

The sample size was 100 patients which was restricted due to the available patient pool during the study period.

Data collection

Data collection commenced from the initiation of feeding and continued until the patient achieved seven consecutive days of full feeds or was discharged from the PICU. Comprehensive anthropometric measurements were recorded for each participant, and nutritional assessments were conducted following WHO guidelines [6]. Calorie requirements were calculated using the Schofield (weight) formula. We documented the timing of enteral feed initiation following PICU admission. The type of feed, its starting rate, advancement of schedule, and time to reach full feeds (prescribed energy goals) were noted. Instances of enteral feeding interruptions, their number, the duration of each interruption episode, and its causes were noted. Causes of EN interruption were categorized into the following broad groups: (a) patient-related factors like hemodynamic instability, significant upper gastrointestinal bleeding, postoperative ileus, and intestinal obstruction and (b) procedure-related factors like endotracheal intubation or extubation, diagnostic tests or procedures in the radiology suite, other procedures done at the bedside or in the operating room, and feeding intolerance. Enteral feeding was held for six hours before elective anesthesia for general surgery or sedation for procedures, and it was held for four hours before elective endotracheal intubation and extubation. If enteral feeding was stopped for more than this period, it was considered an interruption. Two independent pediatricians analyzed the causes of feeding interruptions, categorizing them as avoidable or unavoidable based on their clinical judgment.

Statistical analysis

Upon completion of data collection, the collected data were entered into an Excel spreadsheet (Microsoft Corporation, Redmond, WA) and a master chart was prepared. Subsequently, statistical computations were performed to derive summary statistics. For continuous variables, statistical parameters such as the mean, median, and standard deviation were calculated and reported as applicable. In the case of categorical variables, the analysis involved the determination of both counts and percentages.

Ethical considerations

The MGM Medical College and Hospital's ethics committee approved the study for research on human subjects (vide letter number: MGM-ECRHS 2019/60). Written and informed consent was obtained from the parents of study participants before enrollment.

## Results

The study comprised 100 patients, with a gender distribution of 1:1.2 (male-to-female ratio) and a median age of 27 months. The median length of PICU stay was 5.5 days (Table 1). Only 34% of patients had normal nutritional status upon admission, while the remainder presented with varying degrees of malnutrition. The reasons for PICU admission included neurological illnesses (28%), respiratory problems (22%), surgical issues (18%), and congenital heart diseases (8%) (Table 2).

**Table 1:** Baseline characteristics ^1^Median, PICU: Pediatric Intensive Care Unit

Baseline characteristics	Values
Age^1^ (months)	27
Male-to-female ratio	1:1.2
Weight^1 ^(kg)	5
Height^1^ (cm)	58
Length of PICU stay^1^ (days)	5.5

**Table 2:** Clinical categories

Category	Percentage
Respiratory illness	22%
Neurological disorders	28%
Dengue fever	9%
Neurosurgery/trauma	10%
Gastrointestinal surgery	8%
Congenital heart defect	8%
Haemato-oncology	3%
Sepsis	12%

In 65 patients, nutrition was initiated within 48 hours of PICU admission, whereas in 35 patients, it was initiated after 48 hours. The median time from PICU admission to the initiation of EN was 32.5 hours. Delayed enteral feeding was primarily attributed to hemodynamic instability and the presence of gastric aspirates.

A total of 484 interruptions to enteral feeding were identified, with at least one interruption occurring in 42% of patients during their admission. Enteral feeding was interrupted for various reasons in 42% of patients, with a cumulative interruption duration of 4,096 hours and a median interruption duration per patient of 40.96 hours. The most common reasons for enteral feeding interruptions were altered sensorium (1,226 hours) and respiratory distress (1,008 hours), accounting for 54.8% of the total interruption hours. Other contributing factors included the presence of gastric aspirates (784 hours), radiological investigations (326 hours), and surgical procedures (584 hours) (Table 3). Upon evaluation by two independent pediatricians, it was determined that 76% of these interruptions were avoidable, while 24% were unavoidable. Approximately 56.7% of patients achieved the prescribed energy goal during their PICU stay, with a mean time of 3.71 days to reach 50% of the calorie goal, 4.8 days to reach 70%, and 5.93 days to attain 100% of the calorie goal.

**Table 3:** Interruptions to EN

Causes of interruptions	No. of hours (percentage)
Altered sensorium	1,226 (30%)
Respiratory distress	1,008 (25%)
Gastric aspirates	784 (19%)
Diagnostic procedures	168 (4%)
Radiological investigations	326 (7%)
Surgical procedures	584 (14%)
Total	4,096 (100%)

## Discussion

Despite EN being the preferred mode of nutrient delivery in critically ill patients, barriers to its optimal administration at the bedside persist. A significant proportion of eligible patients experience deprivation of EN during critical illness due to preventable factors, including suboptimal prescription practices, delayed initiation of EN, and frequent and prolonged interruptions to enteral feeding [5,7-9]. Delayed initiation and subsequent interruptions constitute the primary contributors to suboptimal EN delivery in the PICU.

Current clinical guidelines for nutritional support in critically ill children recommend early initiation of EN within the first 24-48 hours following admission [8-10]. However, our study revealed that only a minority (35%) of patients received EN within 48 hours of admission, despite evidence that early EN can improve clinical outcomes, reduce infection rates, shorten hospital stays, and prove cost-effective [11-13]. The delay in EN delivery can be attributed to various factors, including gastrointestinal dysfunction, elective interruptions for procedures, and a lack of awareness among physicians regarding its benefits [13,14]. In addition, some physicians may be cautious about starting EN in critically ill patients due to concerns about potential mesenteric ischemia and non-occlusive bowel necrosis, particularly in hemodynamically unstable patients [15,16]. In our study, the most common reason for delayed EN initiation was hemodynamic instability.

Interruptions in EN are a recognized challenge in critical care settings [17-19]. The care of critically ill patients necessitates multiple interventions that may compete with the delivery of EN in the intensive care environment. Elective procedures, unplanned interventions, and diagnostic tests often require fasting, leading to interruptions in EN. Additionally, feed intolerance or contraindications related to the disease process may necessitate postponing or discontinuing enteral feeding in the PICU. In our study, a notable proportion of patients encountered interruptions in enteral feeding, resulting in a cumulative interruption duration of 2,048 hours. The median duration of interruption per patient was 40.96 hours. The primary causative factors for these interruptions were identified, with the most prevalent being hemodynamic instability attributed to altered sensorium (30%) and respiratory distress (25%). Furthermore, a comprehensive survey conducted across PICUs in the United Kingdom, involving input from PICU physicians, nurses, and dieticians, shed light on significant impediments to enteral feeding. It was observed that 24% of these interruptions were related to patients being deemed "too ill" or experiencing hemodynamic instability [19]. This underscores the significance of hemodynamic instability as a pivotal factor contributing to the interruption of enteral feeding practices. Notably, we found a substantial proportion of these interruptions (74%) were deemed to be avoidable, accentuating the potential for improvement in clinical management.

Mehta et al. conducted a prospective observational study involving 117 children and found that interruptions to EN occurred in 30% of PICU patients, with 58% of these interruptions classified as avoidable. Reasons for avoidable interruptions were feed intolerance, feeding tube issues, around intubation or extubation, and fasting for surgical or radiological procedures [17]. A Canadian survey involving physicians and dieticians also identified fasting for procedures as a major barrier to EN [18]. Fasting for procedures, both within and outside the PICU and in transit to the operating department, poses a considerable challenge for most intensive care patients. In our study, if enteral feeding was withheld for more than six hours prior to elective anesthesia for general surgery or sedation for procedures and for more than four hours prior to elective endotracheal intubation and extubation, it was classified as an avoidable interruption. Currently, there is no established evidence regarding "safe" fasting durations for critically ill children or specific procedures requiring fasting. The fear of aspiration pneumonia during emergency reintubation (in the event of endotracheal tube dislodgment) drives the practice of fasting [19].

It is imperative to acknowledge the limitations inherent in this investigation. This study was conducted at a single center, which inherently restricts the generalizability of our findings to a broader patient population. The relatively small sample size employed in this study underscores the need for caution when extending these findings to more extensive patient cohorts. Furthermore, a notable limitation pertains to the inability to measure resting energy expenditure in PICU patients, primarily due to the unavailability of the requisite facilities. Consequently, estimated caloric and volume goals were employed to prescribe caloric or volume intake, introducing a potential source of imprecision in our assessments. Future investigations should aim to address these limitations to foster a more comprehensive understanding of the intricate interplay between nutrition and clinical outcomes in the context of pediatric critical care.

## Conclusions

This study sheds light on the intricate challenges associated with nutritional management for critically ill children. It underscores that achieving nutritional requirements in pediatric critical care is an infrequent occurrence, with delayed initiation and frequent interruptions being the primary culprits. Importantly, a significant proportion of these challenges are preventable. The utilization of local feeding guidelines, with or without the involvement of nutrition support teams, has proven effective in promoting EN and should be encouraged. A concerted effort involving physicians, nurses, and dieticians is essential to actively address these barriers within the PICU.
